# Functional outcome after digit replantation versus amputation

**DOI:** 10.1186/s10195-022-00654-7

**Published:** 2022-07-27

**Authors:** Sarah M. Bott, Katarzyna Rachunek, Fabian Medved, Thomas S. Bott, Adrien Daigeler, Theodora Wahler

**Affiliations:** 1 Department of Hand, Plastic and Reconstructive Surgery, BG Trauma Center, Eberhard-Karls University Tuebingen, Schnarrenbergstr. 95, 72076 Tuebingen, Germany; 2 Department of Pediatric Surgery, University Hospital of General and Visceral Surgery Freiburg, Hugstetter Straße 55, 79106 Freiburg, Germany; 3 Department of Hand, Plastic and Aesthetic Surgery, Medius Clinic Nürtingen, 72622 Nürtingen, Germany

**Keywords:** Digit, Replantation, Amputation, Functional outcome, Sensitive recovery

## Abstract

**Background:**

The success of digit replantation is mainly based on survival rates. The functional outcome as well as the recovery of sensibility are essential parameters for judging the outcome after digit replantation but have been poorly assessed in previous studies.

**Patients and methods:**

Forty-eight patients with 56 complete traumatic digit amputations occurring between 2008 and 2013 returned for a follow-up examination, the earliest being 6 months postoperatively. Each patient’s range of motion, fingertip-to-table distance, fingertip-to-palm distance, grip and pinch strengths, static two-point discrimination (2-PD), and Semmes–Weinstein monofilament (SWM) test level were assessed in order to compare functional outcome and recovery of sensibility between successful replantation (*n* = 19) and primary or secondary amputation (*n* = 37). Subjective assessments of the pain level and function of the upper extremity were performed using the numerical rating scale and the DASH score, respectively.

**Results:**

Replanted digits achieved 58% of the median total range of motion of the corresponding uninjured digits. Grip and pinch strength were not significantly different after thumb or finger replantation or amputation. Recovery of sensibility was excellent after replantation, with a median static 2-PD of 5 mm and a reduction of pressure sensibility of two levels of the SWM test compared to the contralateral side. After amputation, the median static 2-PD was also very good, with a median value of 6 mm and a reduction of pressure sensibility of only one level according to the SWM test. There was significantly less pain after replantation at rest (*p* = 0.012) and under strain (*p* = 0.012) compared to patients after amputation. No significant differences were observed in the DASH score between the two groups.

**Conclusion:**

Comparable functional results and sensory recovery but significantly less pain at rest and under strain can be expected after digit replantation when compared to digit amputation.

**Level of evidence:**

IV.

## Introduction

Malt's group performed the first successful arm replantation in 1962 [1]. In July 1965, Tamai and Komatsu et al. performed a replantation of a completely separated thumb [2]. Meanwhile, limb replantations are being performed worldwide, and standardized surgical techniques have been developed to obtain better results [3]. Each patient's age and general condition play a role in the decision to attempt a replantation. Amputations that are proximal to the flexor digitorum superficialis (FDS) tendon’s insertion have resulted in poor functional outcome in previous studies [4]. The preferred management of amputations varies between centers and regions [5]. The best prognosis can be achieved within 6 h after injury, but it seems possible to perform a digit replantation within the first 12 h or even longer, especially when cold ischemia of the amputated part is performed [6]. Whether or not a single amputation of a finger should be replanted has been discussed as a controversy. Contraindications for replantation are mostly a long ischemia time, a missing amputated part, relevant loss of tissue due to injury mechanism, a manual occupation, and severely contaminated wounds [7].

However, the success of digit replantation is no longer only defined by the survival rate, since the final functional outcome and the return of sensibility [8, 9] may be even more important. An opposable thumb is essential for hand function, so its replantation is always performed if possible [10, 11]. In prior studies, functional results have been mostly reported after thumb replantation [12–14]. Larger multicenter studies or meta-analyses comparing digital replantation versus revision amputation were recently performed and were based on patient-reported outcomes assessed by questionnaires [15–17]. So far, only a few studies have compared functional outcome and recovery of sensibility between digit replantation and amputation [14, 18].

The goal of this study was to compare the final outcomes of digit replantation and amputation in terms of function, sensory recovery, and pain level.

## Patients and methods

Patients that had at least one traumatic digit amputation between 2008 and 2013 in our trauma center and understood the background of the study were invited for a follow-up examination 6 months after replantation at the earliest. In total, 112 patients suffered 152 traumatic digit amputations during this period. Replantation of 80 digits was attempted in 73 patients. Secondary amputation had to be performed in 36 digits after a mean time of 9 days (range 2–20 days). Primary amputation was performed in 72 digits. The study included all patients with complete amputations as defined by Biemer [19]. The study excluded subtotal amputations. Two patient groups were formed: those after a successful replantation (group R) and those after primary and secondary amputation (group A). Primary amputation was performed due to different reasons such as a missing or destroyed amputated part or if the patient rejected a replantation attempt. Furthermore, secondary amputation was performed after a replantation attempt had to be aborted due to vascular failure.

**Fig. 1:**
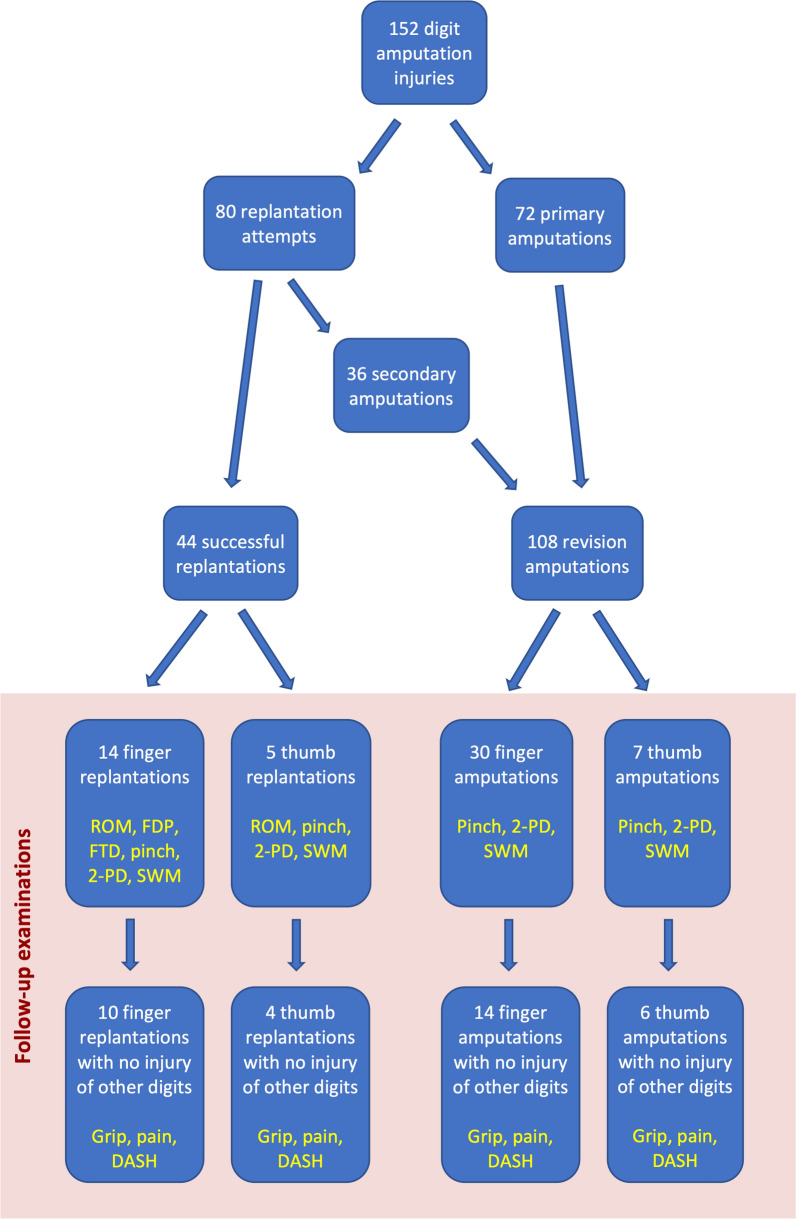
Flowchart depicting the distributions of patients and digits according to the different assessments during follow-up examination. Tests concerning the function of the whole hand and not of a single digit, such as grip strength, pain levels, and DASH score, were performed in patients with a single-digit injury only

Forty-eight out of the 112 patients with 56 complete digit amputations returned for a follow-up examination (Fig. 1). They each gave written consent to participate in the study. Thirty-four patients had a single traumatic digit amputation only, seven had a single digit amputation and an additional subtotal amputation of another digit, and seven had multiple digit amputations (six with two- and one with three-digit amputations). Nineteen patients were examined after 14 successful finger replantations and five successful thumb replantations (6F, 13M, mean age 44.7 years). Thirty-one patients were examined after 30 finger and seven thumb primary or secondary amputations (6F, 25M, mean age 44.6 years). Two patients with a single successful replantation also had one or two amputations of other digits, respectively. Six further patients had amputations of two digits of the same hand. The local ethical committee (project number 006/2013B02) approved the study.

### Surgical treatment

The operation occurred with axillary plexus block anesthesia in most of the cases. The surgeons prepared the amputated digit by seeking and marking digital arteries, nerves, and veins during preoperative preparation of the patient. The operation began with bone stabilization using K-wires and cerclages, followed by flexor and extensor tendon repair. Six patients needed arthrodesis after finger amputation (four of the proximal interphalangeal (PIP) and two of the distal interphalangeal (D﻿IP) joint) and two needed it after thumb replantation (one of the interphalangeal (I﻿P) and one of the metacarpophalangeal (MCP) joint). Subsequently, arteries, nerves, and finally veins were reconstructed. After replantation, the patients were put on a 15,000 unit heparin perfusion system 24 h a day for five days. An intravenous antibiotic therapy with mostly a first-generation cephalosporin was administered for about seven days. Patients were on strict bedrest for between three and 14 days.

During primary or secondary surgical amputation, an effort was made to maintain the maximum possible stump length with sufficient distal soft-tissue coverage. Care was taken to selectively shorten the digital nerves by about 2 cm in order to avoid neuroma formation close to the new fingertip.

### Functional outcome

**Fig. 2:**
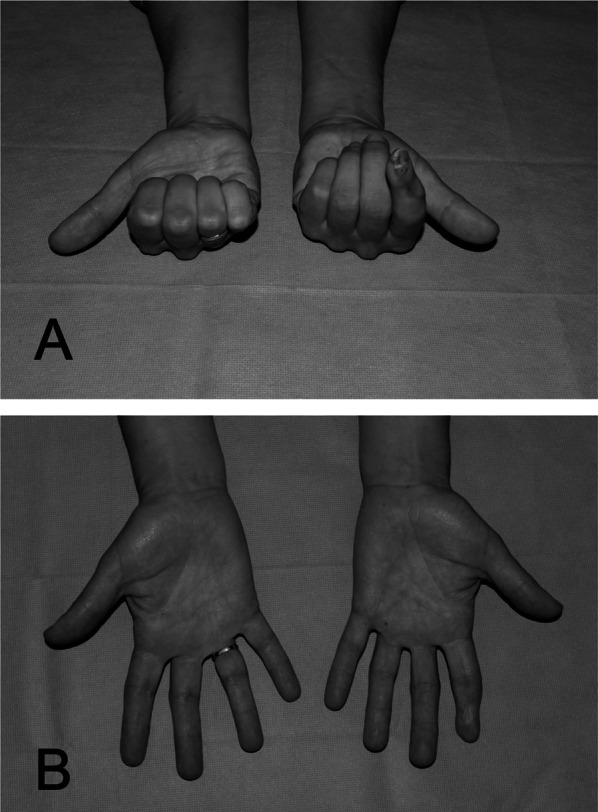
Photographs showing the examination of the fingertip-to-palm distance (FPD, **A**) and the fingertip-to-table distance (FTD, **B**)

A goniometer determined the range of motion (ROM) of the metacarpophalangeal and interphalangeal joints of the replanted digits [20]. The fingertip-to-palm distance (FPD) [21] and fingertip-to-table distance (FTD) were used to assess deficits in finger extension and flexion of replanted digits (Fig. 2).

The Biometric E-Link Evaluation System (Biometrics Ltd, Newport, UK) was used to determine the grip and pinch strengths. Grip and pinch measurements were taken in the standardized sitting position with the shoulder of the patient kept in the neutral position and the elbow flexed to 90° [22]. The grip strengths of patients with single digit replantation (*n* = 14) or amputation (*n* = 20) were recorded in the standardized level II position. Multiple digit injuries and partial injuries without complete amputation of the digit were excluded from the grip testing, since a lower force is expected in those cases. This is not the case for the pinch test, since the thumb is opposed only to a single finger. For this examination, multiple digit replantations were also included. Each injured digit’s pinch strength was measured using a pinchmeter to detect the force between the thumb and the injured digit compared with the contralateral side [20]. If the thumb was affected, the force was measured between the thumb and the next uninjured digit (mostly the index). The average grip and pinch strengths of three trials were recorded.

### Recovery of sensibility

The static two-point discrimination (2-PD) and Semmes–Weinstein monofilament test (SWM test) were used to evaluate the sensory recovery of the radial and ulnar digital nerves of all injured fingers. Normal sensibility was defined as the sensibility assessed at the corresponding contralateral uninjured digits. The two-point discriminator consisted of a plate with several pins with adjustable separations ranging from 1 to 15 mm in steps of 1 mm. The patients were asked to close their eyes during the examination. One or two pins were applied perpendicularly to the radial and ulnar finger pulp and held for at least 3 s. In the case of amputated digits, the examination took place on the radial and ulnar palmar sides of the distal stump, as similarly performed on an intact digit. Three repeated responses were necessary for the final score [23].

The Homecraft Rolyan SWM test set contains 20 nylon monofilaments with different diameters [23]. For better handling, each monofilament was fixated at a 90° angle on a plastic pen. The patient was asked to close their eyes while the monofilament was applied perpendicularly on the radial and ulnar finger pulp until it bent, holding it for 1–2 s. The testing began with the smallest monofilament, followed by the next monofilament size if the patient was not responding. The uninjured side was tested and compared to the injured side in order to calculate the reduction of sensibility in level difference, meaning the number of ordinally ordered monofilaments between the injured and uninjured sides. In the case of amputated digits, the examination took place on the radial and ulnar palmar sides of the distal stump.

### Pain level and DASH score

The numerical rating scale (NRS) and the disability of the arm, shoulder, and hand (DASH) questionnaire were used for subjective evaluations. Only patients with a single-digit injury were included for these evaluations. This is because patients with multiple-digit injuries are expected to have a higher pain level and a higher DASH score than patients with a single-digit injury and their inclusion would, therefore, bias the results. On a scale from 0 to 10, the pain intensity at rest, under strain, and in cold temperatures was reported, with a higher number indicating increased pain [24].

The DASH questionnaire contains 30 questions on daily living activities for assessing the patient’s disabilities in the upper extremities. Each question is scored on a five-point scale from 1 to 5. The score calculation ranged from 0 to 100, while a higher DASH score indicated increased disability [25]. At least 27 of the 30 items were needed to obtain a valid score.

All tests as performed in different patient populations are summarized in Fig. 1.

### Statistical analysis

Statistical analysis was performed using SPSS version 26.0.0 (IBM; Armonk, New York, USA). Pie charts depicted the finger distributions in both groups. Boxplots with median and interquartile ranges were created to graphically display the measured values of the 2-PD, SWM test, NRS, and DASH score. Data beyond these bars were depicted as outliers. The results for the grip and pinch strengths, 2-PD, SWM test, NRS, and DASH score were evaluated for statistical significance. Due to a non-normal distribution, the Mann–Whitney *U* test was used to define probabilities of statistical significance between the groups. A *t* test was performed in the case of a normal distribution. Results were considered significant when *p* < 0.05.

## Results

The follow-up examination took place 6–76 months (median 32 months) after surgery.

**Fig. 3:**
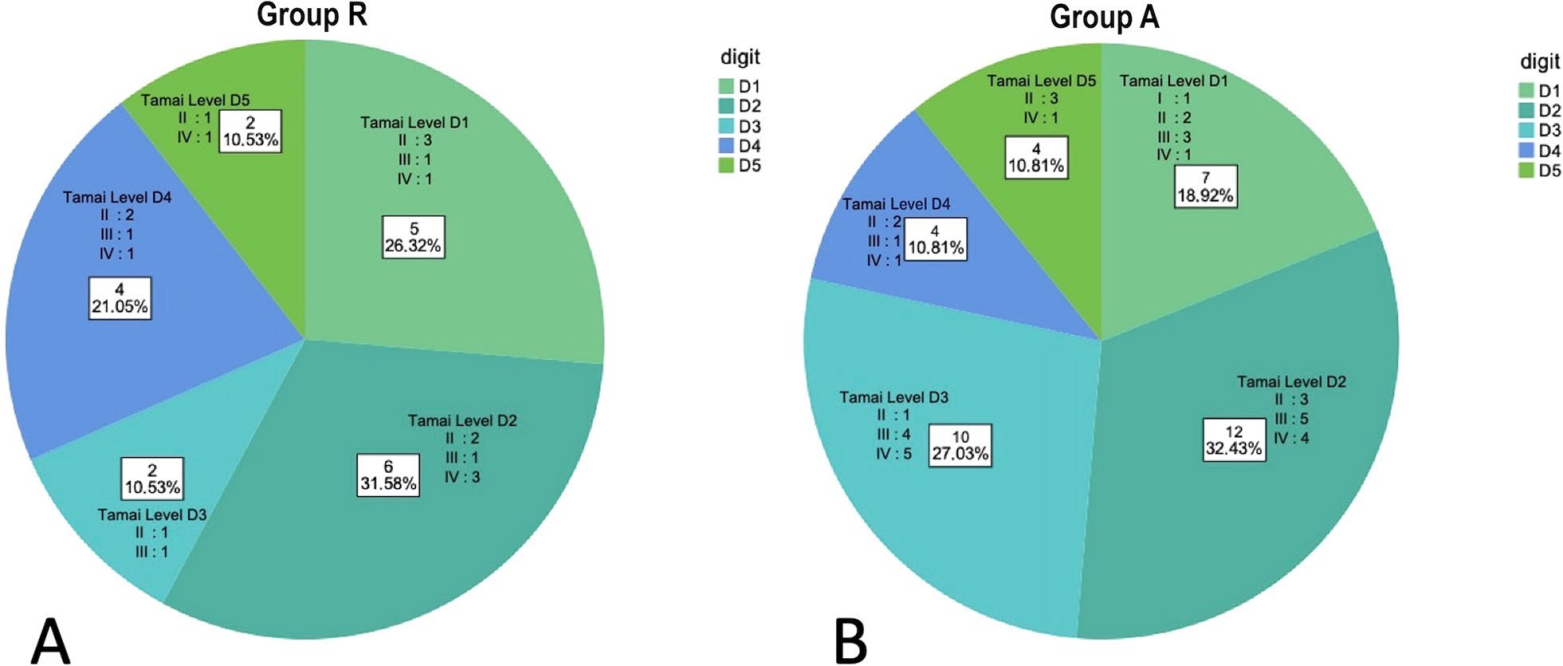
Pie charts depicting the finger distributions, including the Tamai level of injury, in group R (**A**) and group A (**B**).* D1* thumb,* D2* index,* D3* middle,* D4* ring,* D5* small finger

Pie charts depicting the digit distribution and the Tamai level of amputation [26] in both groups are shown in Fig. 3. The most affected digit was the index in both groups, followed by the thumb in the replantation group. The middle finger was more commonly subjected to surgical amputation than replantation.

### Motion of replanted digits

Measurements of the range of motion (ROM) including the MCP and IP joints of the 14 replanted fingers averaged 146° (range 80–195°). The median ROM achieved was 148°. Compared to the uninjured side, the ROM averaged 241° (range 151–286°, median 255°). After finger replantation, the ROM was 58% compared to the uninjured hand. The average ROM of the five replanted thumbs (MP and IP joints) was 91° (range 43–151°, median 87°), compared to the contralateral uninjured thumbs with an average ROM of 144° (range 84–174°, median 149°). The ROM of the thumb after replantation was 58% compared to the uninjured hand.

The FTD and FPD were assessed in 14 patients after finger replantations. Patients with thumb amputations were excluded from this measurement. The median FTD of the replanted fingers was 0.75 cm and the median FPD was 2 cm.

The ROM, FTD, and FPD in the amputation group were not measured due to the shortened digit length and, therefore, noncomparable results.

### Grip and pinch strength

The grip strength could be evaluated in all 14 patients of group R and all 20 patients of group A with single-digit amputation. After finger replantation (*n* = 10), the healthy hand could achieve a mean force of 78.7%, compared with 87.9% after finger amputation (*n* = 14). A mean force of 91.5% after thumb replantation (*n* = 4) and 66.0% after thumb amputation (*n* = 6) could be achieved in terms of grip strength compared to the contralateral side. The above differences in grip strength between group R and group A after thumb or single-finger traumatic amputation were not significant.

The pinch strength could be measured for 16 of the 19 digits in group R and 35 of the 37 digits in group A due to a technical problem during the examination of the missing cases. After finger replantation (*n* = 11), 78.7% of the strength of the healthy hand could be achieved, compared with 87.9% after finger amputation (*n* = 29). The pinch strength of the injured thumbs reached a mean force of 60.4% in group R (*n* = 5) and 61.3% in group A (*n* = 6) compared with the contralateral uninjured digits. The above differences in pinch strength between group R and group A after thumb or single-finger traumatic amputation were not significant.

### Recovery of sensibility

**Fig. 4:**
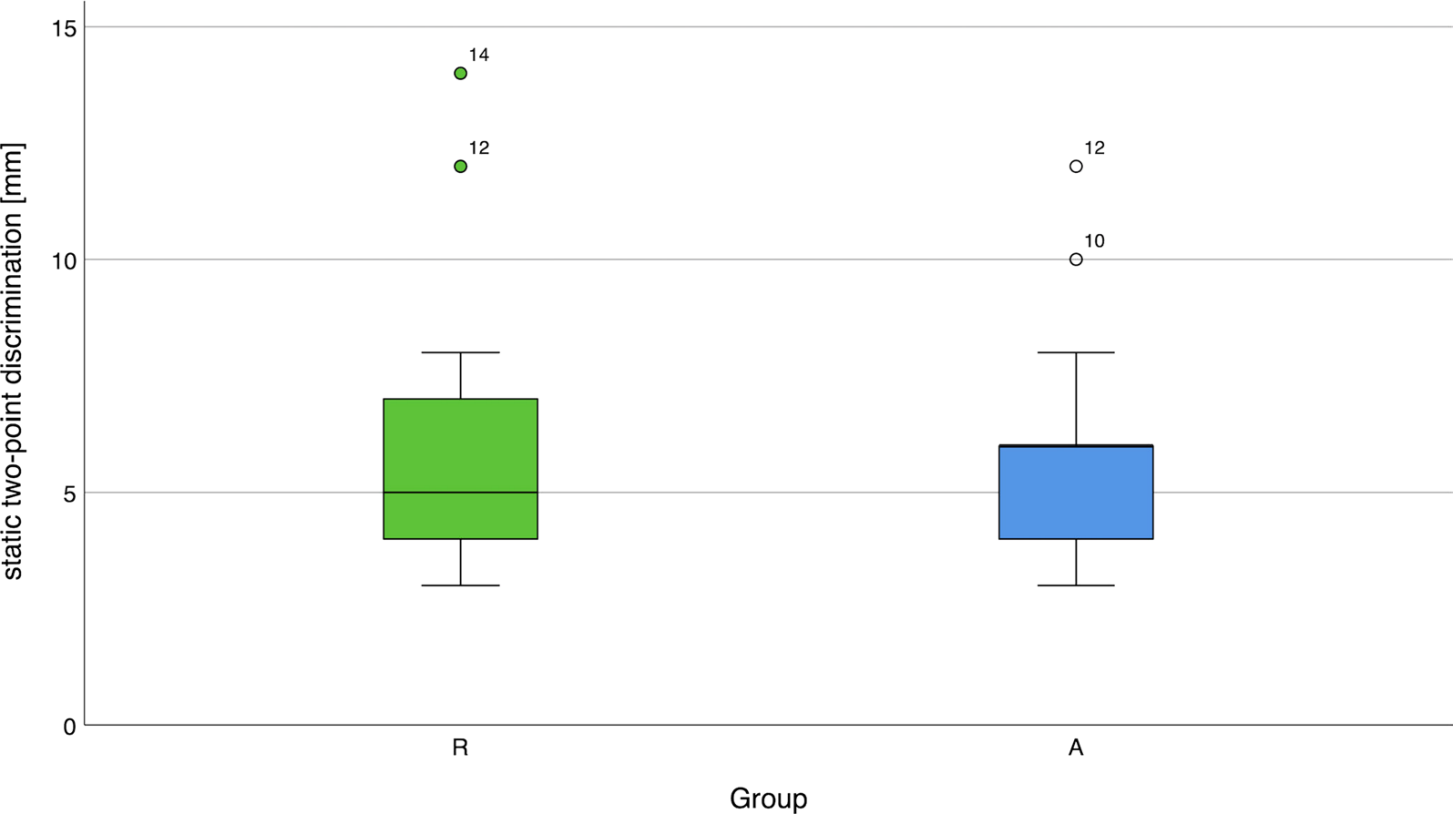
Boxplots comparing the static two-point discrimination in groups R and A.* Whiskers* extend to a maximum distance of 1.5 interquartile ranges. Outliers are beyond these bars

The static 2-PD (< 15 mm) was recovered in 29 out of the 33 reconstructed digital nerves (87.9%) in group R. In group A, it was regained in 66 out of 74 reconstructed digital nerves (89.2%). Both digital nerves in two patients and only the ulnar nerve in one patient could be sutured due to the distal level of injury. Boxplots depicting the results of measurable 2-PD are presented in Fig. 4. The median static 2-PD was 5 mm in group R and 6 mm in group A, and showed no statistically significant difference between the two groups.

**Fig. 5:**
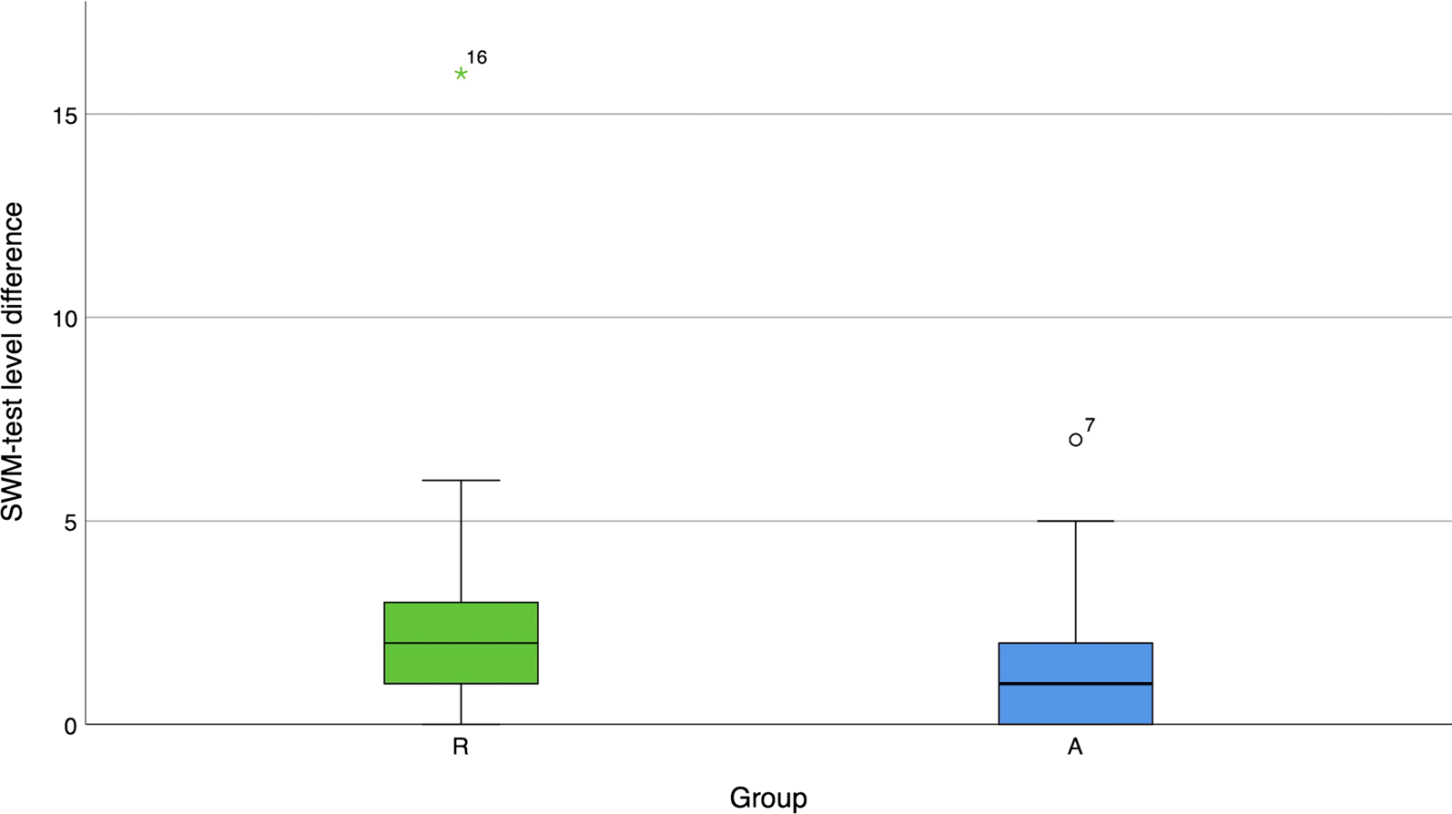
Boxplots comparing the Semmes–Weinstein monofilament level difference between the injured and uninjured sides in groups R and A

The recovered pressure sensibility could be tested using the SWM test in all 107 cases (33 digital nerves in the replantation and 74 digital nerves in the amputation group). Results are depicted in Table 1. Boxplots summarizing the reduction of sensibility at the innervation area of the reconstructed digital nerves, defined as the level difference between the injured and healthy sides, are presented in Fig. 5. A median reduction of sensibility of two levels was observed in group R compared to one level in group A. This difference was statistically significant (*p* = 0.034). In both groups, most reconstructed digital nerves demonstrated axcellent recovery in terms of normal touch or diminished light touch. This occurred in 58% of the cases in group R and 70% of the cases in group A.

**Table 1:** Semmes–Weinstein monofilament test

	Group R	Group A
Number of nerves examined by SWM test	33	74
Normal touch (N) (%)	7 (21.2%)	28 (37.8%)
Diminished light touch (DLT) (%)	16 (48.5%)	24 (32.4%)
Diminished protective sensation (DPS) (%)	8 (24.2%)	20 (27%)
Loss of protective sensation (LPS) (%)	2 (6.1%)	2 (2.7%)

Overview of the number and recovery results of reconstructed digital nerves assessed by the SWM test in the replantation (R) and amputation (A) groups

### Pain level and DASH score

**Fig. 6:**
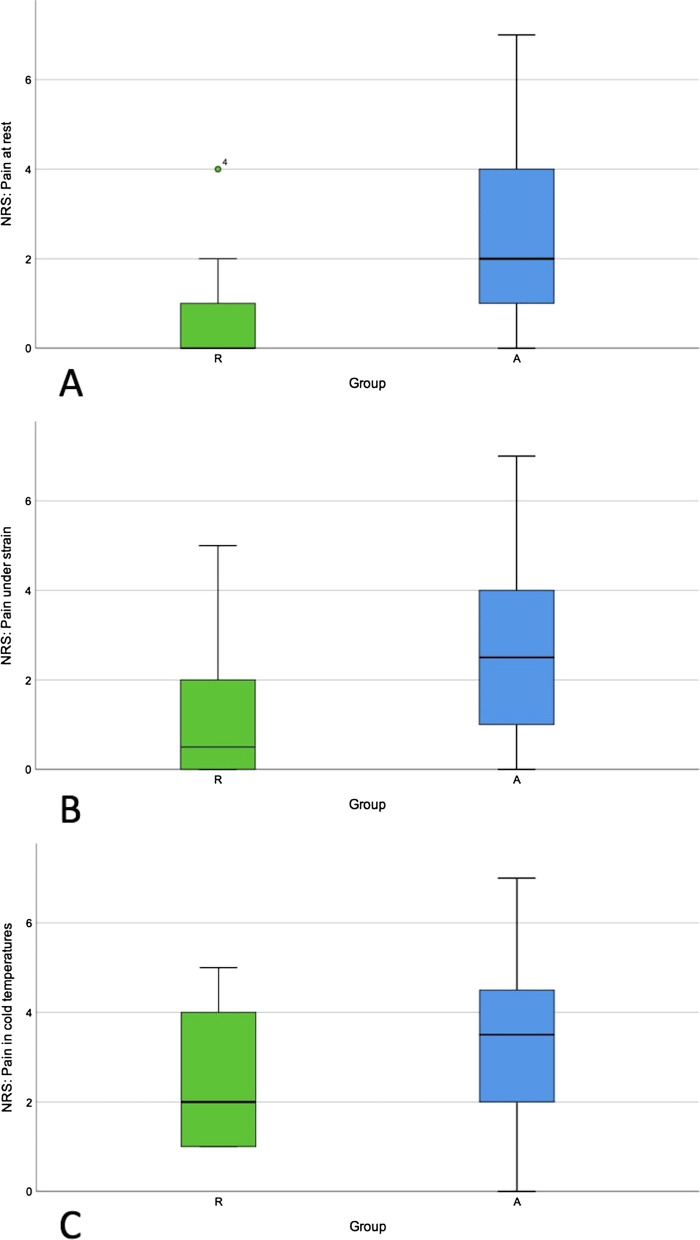
Boxplots depicting the pain intensity according to the numerical rating scale at rest (**A**), under strain (**B**), and in cold temperatures (**C**) in groups R and A

Pain intensity was recorded in all 34 patients with single-digit injuries. Boxplots depicting the pain intensity numerically via the NRS at rest, under strain, and in cold temperatures are presented in Fig. 6. Significantly less pain occurred in the replantation group at rest (*p* = 0.012) and under strain (*p* = 0.012) compared to the amputation group.

**Fig. 7:**
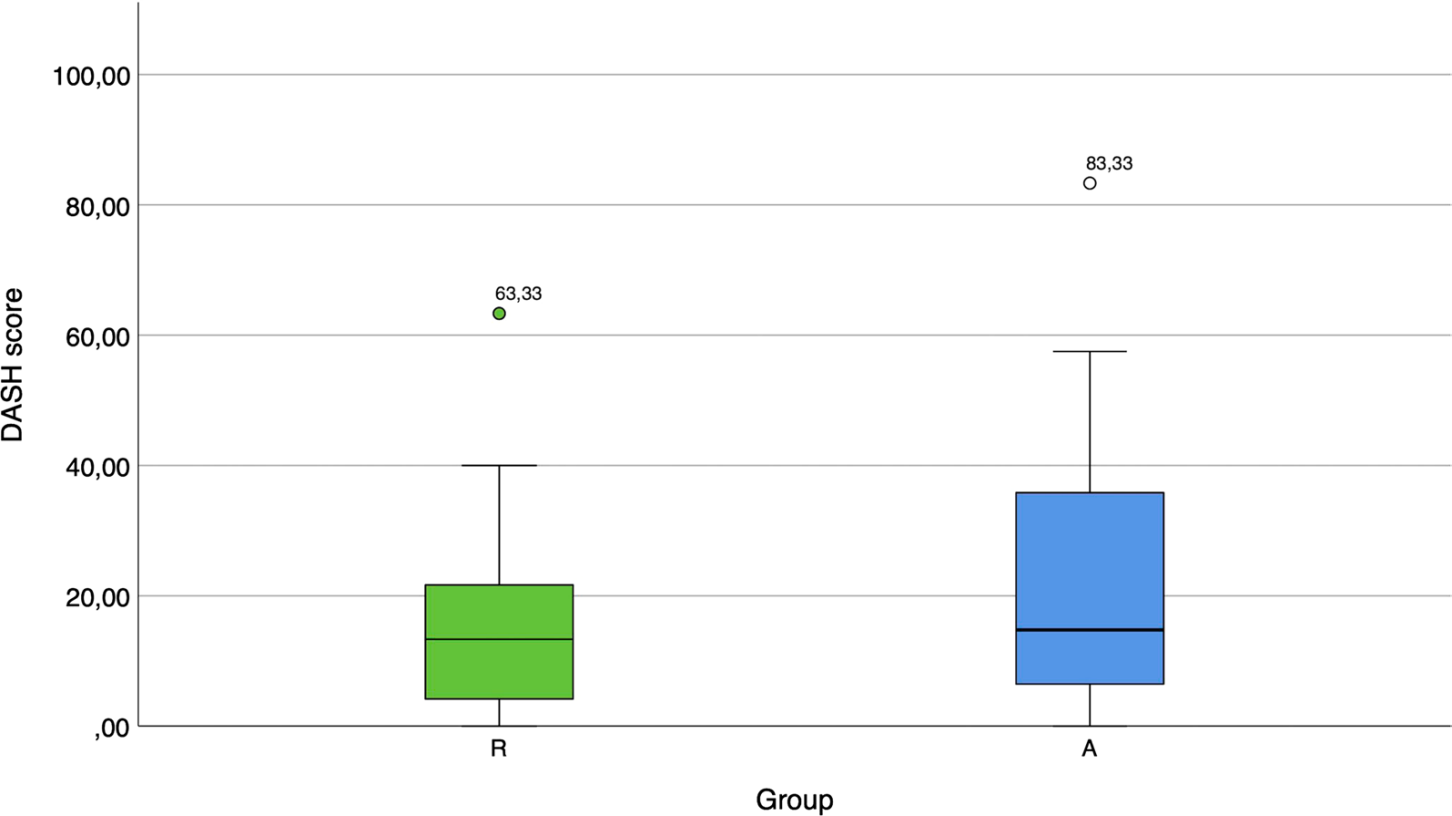
Boxplots presenting the DASH scores in groups R and A

The DASH score was evaluated in all 34 patients with single-digit injuries. The median DASH score in group R (13.33) was lower than that in group A (14.76). Boxplots comparing the DASH scores of both groups are depicted in Fig. 7. No statistically significant difference could be detected between the two groups.

## Discussion

Since the first successful thumb replantation, performed by Komatsu and Tamai in 1965, numerous replantations have been performed, and results have been compared based on the survival rates [2, 8, 9, 11, 12, 27]. However, indications for replantation have also been controversially discussed [27]. Replanted digits often result in stiffness [4, 28], immobility, and cold intolerance [29]. Brown suggested in 1982 that a well-healed stump may be the best solution for many patients [30]. Our study’s aim was to compare the objective and subjective results after replantation versus amputation.

### Functional results (ROM, FTD, FPD, and grip and pinch strengths)

Urbaniak et al. reported a mean ROM of 206° for a replanted finger after avulsion injury [31], and Adani et al. reported a mean ROM of 185° after ring finger avulsion injury [8]. A meta-analysis from 2011 yielded a ROM of 174° after 75 finger replantations [32]. In our study, the mean ROM after finger replantation was less than the results mentioned above (mean 146°). This could be because six of the 14 examined patients with a successful finger replantation had an arthrodesis of either the PIP or DIP joint. After thumb replantation, the mean ROM in this study was 91° (median 87°). In Unglaub et al.’s study, a mean ROM of 56° was found after thumb replantation [13].

In our study population, the median FPD was better than the median FTD. This indicates a greater limitation on finger flexion than extension. Reasons for this could be tendon adhesions and, in some cases, patient compliance during postoperative treatment.

Unglaub et al. described a mean force of 70% in grip strength and 68% in pinch strength after thumb replantation compared to the uninjured side [13]. In this study, a mean force of 92% was achieved in the grip test and 60% in the pinch test after thumb replantation. After thumb or finger replantation, no statistically significant differences in grip or pinch force could be found between groups R and A. It should be kept in mind that differences are expected whether the dominant or nondominant hand is affected [33]. The contralateral uninjured hand served as the control, since amputation injuries are emergency cases and no preinjury assessment of the affected side is possible.

### Recovery of sensibility

In this study, the measurable median 2-PD was 5 mm in group R compared with 6 mm in group A. These are excellent results compared to previous studies. In a meta-analysis in 2011, the results after replanting 2273 digits were evaluated. Sensibility recovery was measured in 12 out of 30 studies and showed a mean of 7 mm [34]. In 2013, Adani et al. reported a mean static 2-PD of 12 mm (range 9–15 mm) after complete avulsion amputation injuries [8]. In contrast to our results, the 2-PD was only described after successful replantation in the previously described publications. Hence, there are no comparable values after amputation.

While the 2-PD measures spatial discrimination, the SWM test quantifies the cutaneous pressure threshold [23]. Criticism of the 2-PD includes the fact that it is a nonobjective test since the force the examiner applied is not standardized [23, 35, 36]. In contrast to the 2-PD, the SWM test is more reliable and objective [35]. Sensibility recovery was mostly tested using the 2-PD test in previous studies. Only one study used the SWM test after 24 thumb replantations [13]. This group reported normal sensibility (N) in one patient (4%), diminished light touch (DLT) in six patients (25%), diminished protective sensation (DPS) in 13 patients (54%), and a loss of protective sensation (LPS) in three patients (12%). Significantly worse results were present in patients with crush and avulsion injuries [13]. In comparison, better results were present in our replantation group. Normal sensibility was present in 21.2%, DLT was found in 48.5%, 24.2% showed DPS, and only 6.1% had LPS.

The superior sensory results in this study may be due to the progress made in microsurgical skills in recent decades when compared to older studies. Exact coaptation with two or three epineural 10–0 or 9–0 nylon sutures of the digital nerves is mandatory for obtaining optimal results [35].

### Pain level

Cold intolerance after successful replantation occurred twice as often as in the amputation group in a study published in 1990 [14]. Jones et al. also reported cold intolerance as being the most common symptom after replantation [29]. Our results demonstrated significantly more pain at rest and under strain after revision amputation than after successful replantation. Importantly, most patients reported no pain at rest at all after successful replantation, while 80% of the patients reported a pain intensity at rest ranging from 1 to 7 (median 2) after amputation, according to the NRS.

### DASH score

In the current study, a similar DASH score was obtained for groups R and A. It should be considered that the DASH score has its limitations because it does not differentiate between the dominant and nondominant sides [37]. This could result in a more unsatisfactory outcome if the injury ended in a stump formation but the patient originally wished for a replantation, and vice versa. The fact that the DASH questionnaire contains questions concerning the whole upper extremity and not just the hand function must also be kept in mind.

### Current aspects of digit replantation

Comparing our overall results with previous studies, it is notable that functional studies assessing diverse parameters, such as the present work, are rare in the literature. For instance, ROM was regularly reported in older studies [8, 32], but grip and pinch strengths were mainly reported after thumb replantation [13]. The excellent sensory recovery results in the replantation group but also the very good remaining sensibility at the stump sites are probably due to the evolution of microsurgical skills, as mentioned above. Similar comparisons of sensory results not only in replanted but also in amputated digits are scarce in the literature. In contrast to older studies (more than 20 years old) [14, 29], pain levels were significantly less in the replantation group, again illustrating the evolution in microsurgical techniques that has occurred in the last decade. Due to meticulous nerve coaptation and anastomosis of both digital arteries and at least two veins in most of the cases, less pain due to neuroma formation or ischemia is expected.

A recent problem in Western and especially American countries is a declining replantation rate, resulting in less experience in digit replantation among surgeons and worse results concerning replantation success. Some explanations for that could be a change in workplace safety, injury characteristics, economic aspects, the surgeon’s attitude, and a lack of technical skills [38, 39]. This is the reason why actual studies examining functional results after digit replantations became rare in the last decade. Our results in a European hand surgery trauma center illustrate the importance of performing digit replantation, if feasible.

### Limitations of the study

The main limitation of the study is the small number of patients, particularly in the replantation group, due to loss to follow-up. However, it is not easy to obtain a larger number of patients who are willing to take part in a follow-up examination in a single-center study years after their treatment. It would be interesting to perform further group comparisons, for example, between primary and secondary amputation, the corresponding digits of each side separately, between injury types, between single or multiple replantations on the same hand, and between dominant and nondominant hands. Furthermore, the assessment of more long-term health-related quality of life outcomes could enrich the comparisons mentioned above [40].

## Conclusion

This study shows that good functional results and an excellent recovery of sensibility can be expected after digit replantation. Compared to digit amputation, similar functional and sensory results can be expected after replantation, in addition to significantly less pain. Surgeons are, therefore, encouraged to perform digit replantation in any reasonable case.
